# Positive Antinuclear Antibody and Blaschkoid Lichen Planus Pigmentosus

**DOI:** 10.7759/cureus.22366

**Published:** 2022-02-18

**Authors:** Amal AlBalbeesi

**Affiliations:** 1 Dermatology, King Saud University College of Medicine, Riyadh, SAU; 2 Dermatology, King Khalid University Hospital, Riyadh, SAU

**Keywords:** connective tissue diseases, antinuclear antibody, lichen planus, ashy dermatosis, blaschko lines, : lichen planus pigmentosus

## Abstract

Lichen planus pigmentosus is a rare variant of lichen planus. It is an acquired pigmentary disorder of unknown etiology. It is characterized by dark brown and slate gray macules and patches. The nails, scalp, and oral mucosa are usually spared, unlike lichen planus. Lichen planus pigmentosus commonly involves the head and neck region as well as intertriginous areas such as the axillae, inframammary and inguinal regions. It can be associated with autoimmune diseases, endocrinopathies, and other variants of lichen planus such as fibrosing alopecia of the scalp. Variable clinical patterns of lichen planus pigmentosus including zosteriform, linear, and segmental had been published. Histopathologically, it is characterized by hyperkeratosis of the epidermis, hypergranulosis, variable degrees of lichenoid infiltration depending on the age of the lesion, and prominent melanin incontinence. Recent updates on erythema dyschromicum perstans that were considered similar to lichen planus pigmentosus, concluded that they could be differentiated on clinical bases as well as histopathology. Epidermal hyperkeratosis, hypergranulosis, apoptotic cells, lichenoid dermatitis, periappendageal infiltrate, and fibrosis with marked superficial dermal melanin incontinence aid to differentiate lichen planus pigmentosus from erythema dyschromicum perstans. During embryogenesis, cells migrate and follow developmental lines named after Blaschko, a German dermatologist, who first noted them. Blaschko’s lines (BL), do not follow neural, vascular, or lymphatic pathways. They appear as V-shaped on the back, S-shaped on the abdomen, and linearly on limbs. We report a case of lichen planus pigmentosus over BL that is a rare presentation of the disease and associated positive antinuclear antibody (ANA) without overt manifestations of any connective tissue disease.

## Introduction

In 1956, Shima was the first to describe lichen planus pigmentosus, and was considered to be a variant of lichen planus. It involves most commonly the face, trunk, and extremities [1]. Infrequently the intertriginous areas are involved. However, antinuclear antibody (ANA) was reported to be positive by indirect immunofluorescence using rat esophagus, in mucosal erosive lichen planus, in 40.42% of patients [2]. Blaschko’s lines (BL) were named after Alfred Blaschko, a German dermatologist in 1858-1922 [3,4]. They do not follow neural, vascular, or lymphatic pathways. The cell migration appears as V-shape on the back, S-shape on the abdomen anteriorly, and as linear lines on the extremities. Congenital and acquired dermatosis have been reported to present over these lines. Linear nevus sebaceous, incontinentia pigmenti, epidermal nevus, and focal dermal hypoplasia (Goltz syndrome) are among the congenital diseases that follow BL [5]. Lichen striatus, lichen planus, psoriasis, atopic dermatitis, graft versus host disease, lupus erythematosus, adult blaschkitis, and lichen nitidus could follow BL [6].

## Case presentation

A 30-year-old female presented to the dermatology clinic with asymptomatic pigmentation over the right side of the trunk for one year following C section for abruptio placenta at 28 weeks, not preceded by erythema. The lesions started on the breast and then spread to involve the chest and abdomen sparing both axillae and face. The patient complained of knee joint pains and oral ulcers prior to the eruption but there was no history of photosensitivity or malar erythema. Also, there was no significant past medical history including autoimmune diseases, drug ingestion, or topical application.

**Figure 1:**
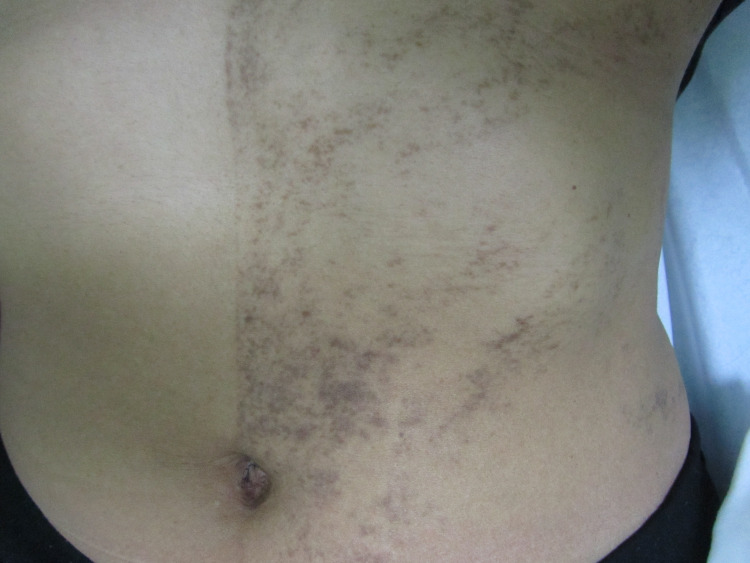
Unilateral violaceous and brown macules forming lines over trunk respecting the midline

**Figure 2:**
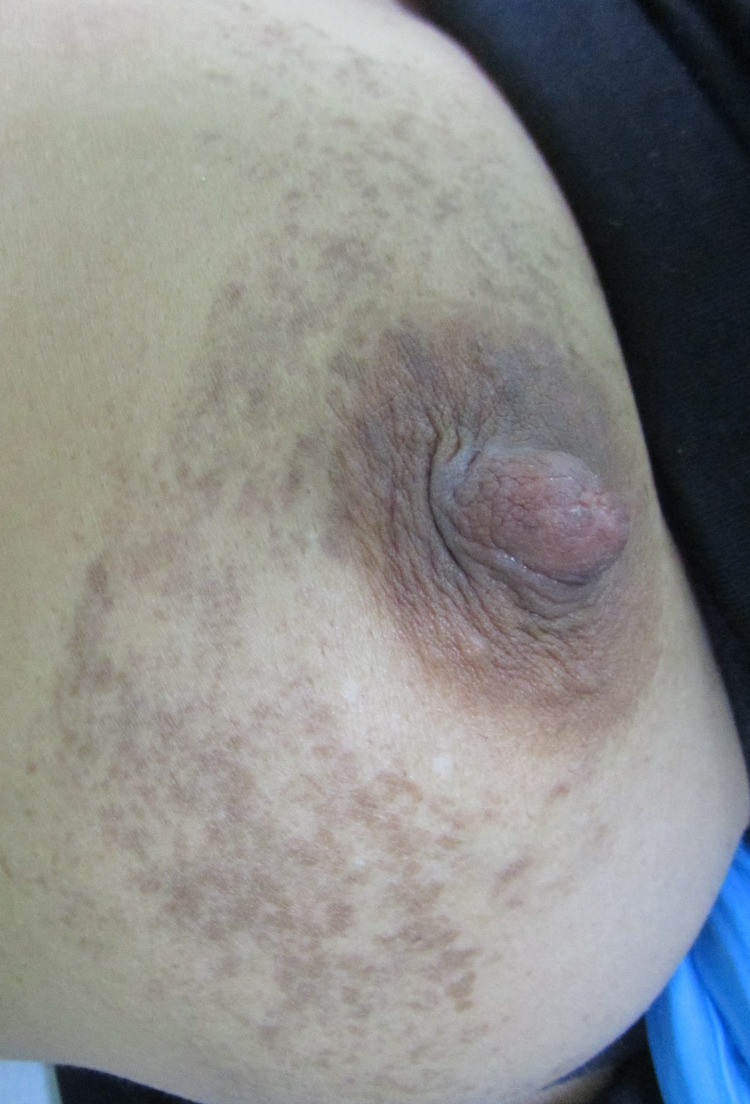
Brown and violaceous macules around breast and areola

**Figure 3:**
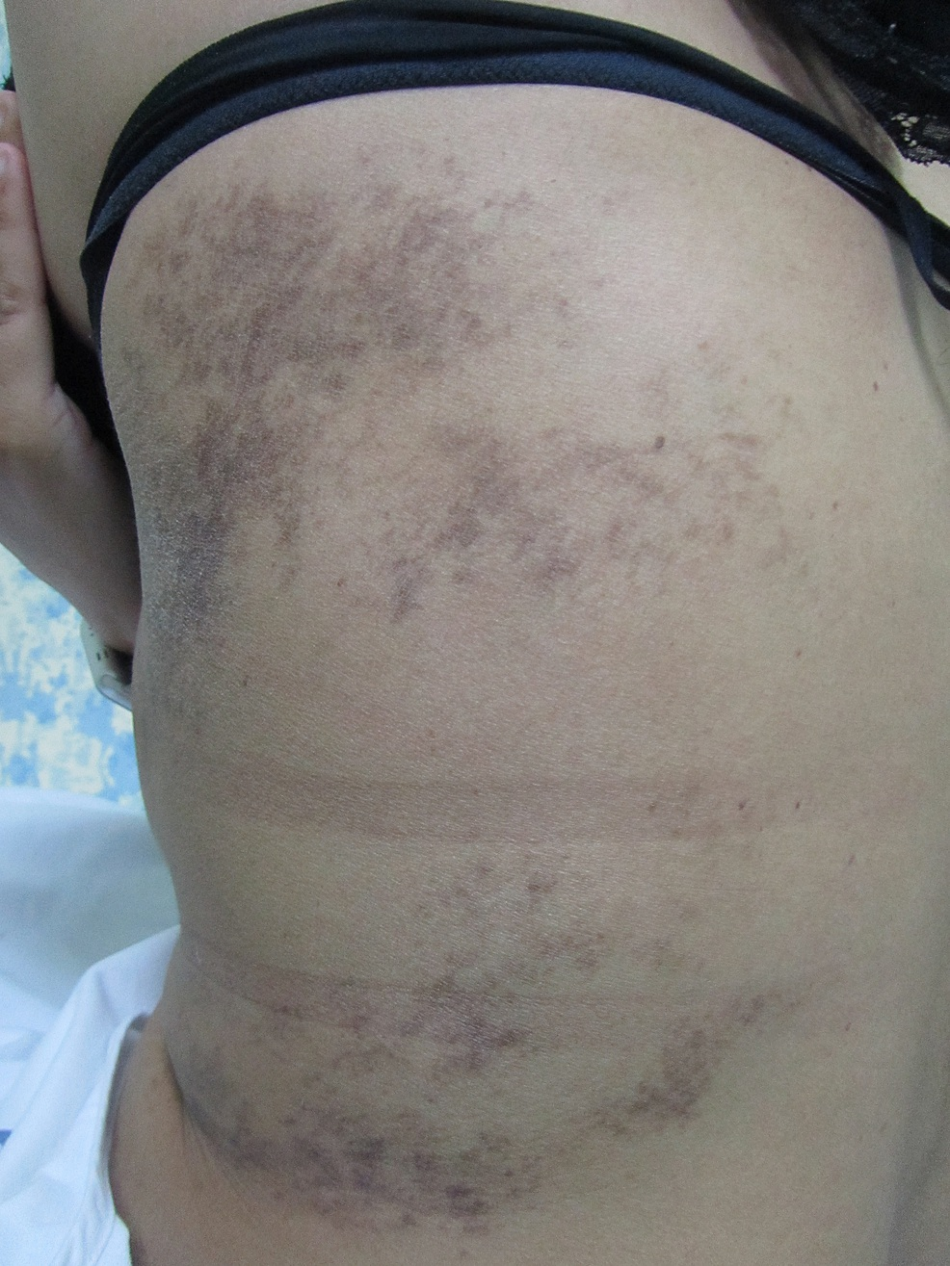
Violaceous blotches and thick lines over the back

On examination, she had non-scaly multiple confluent and reticulated violaceous-brown blotchy macules and lines over breast and trunk that sharply respected the midline following BL (Figures 1-3).

The face, neck, underarms, nails, and mucous membranes were spared. There were no discoid lupus erythematosus (DLE) lesions over the head and neck region. Mucous membrane examination showed gingivitis; however, no ulcers or any oral lesions were seen.

Complete blood count, erythrocyte sedimentation rate (ESR), liver and kidney functions, fasting blood sugar, and thyroid function tests including thyroid-stimulating hormone (TSH) and thyroxine (T4) were all normal.

Hepatitis screening including B and C were negative. ANA titer was 1:1280. However, double-stranded deoxyribonucleic acid (Ds DNA), antiSmith, antiphospholipid antibodies, lupus anticoagulant, Sjogren’s syndrome A autoantibody (SS-A) and Sjogren’s syndrome B autoantibody (SS-B), anti-smooth muscle antibody (ASMA), were all negative. Elevated ANA titer is recognized in rheumatological and non-rheumatological diseases including Hashimoto's thyroiditis and autoimmune hepatitis as well as in the normal population. Based on the normal liver function test (LFT), negative ASMA, and normal thyroid function, the diagnosis of autoimmune hepatitis and thyroid diseases were excluded.

**Figure 4:**
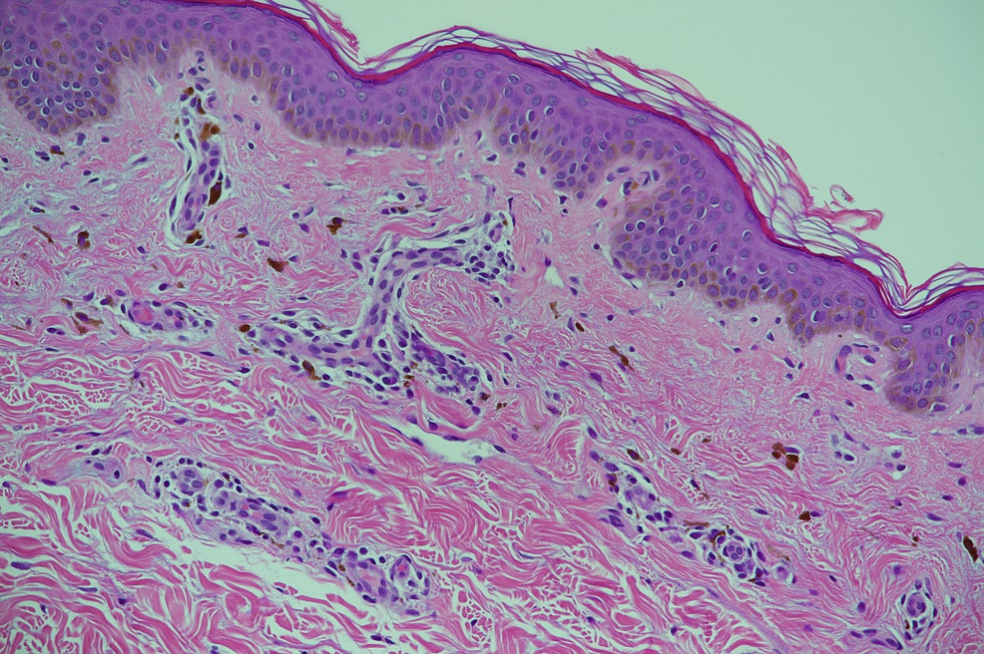
Basket weave stratum corneum, atrophic epidermis, small foci of lymphocytic exocytosis pigment incontinence in the dermis (H&E)

**Figure 5:**
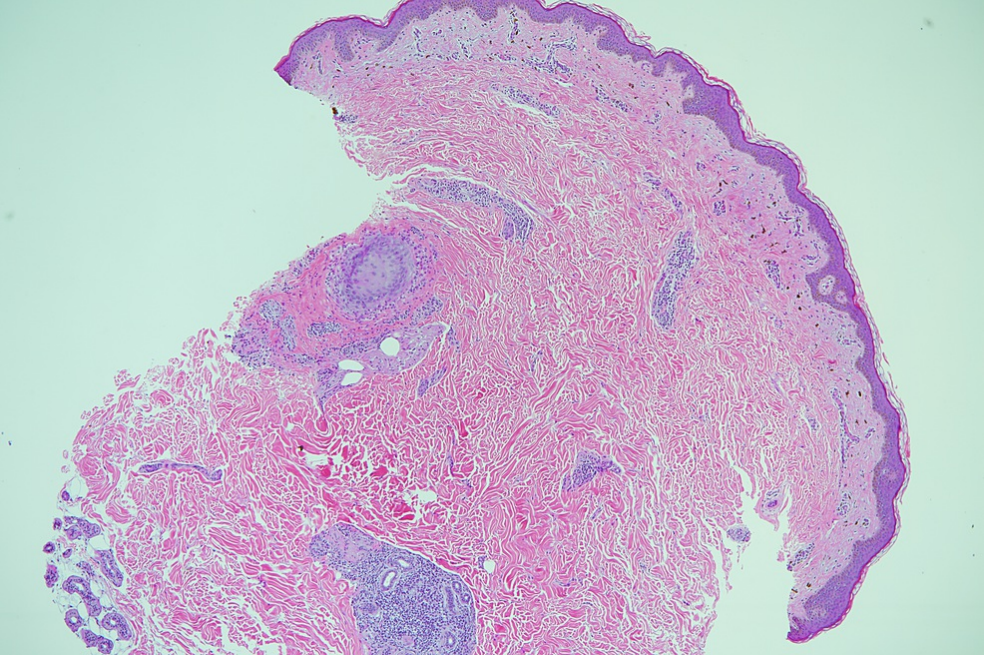
Perifollicular lymphohistocytic infiltrate and fibrosis, mild perivascular infiltrate (H&E)

Histopathological examination of a skin punch biopsy showed basket weave stratum corneum, atrophic epidermis, small foci of lymphocytic exocytosis but no necrotic keratinocytes or vacuolar changes. In the dermis, there was pigment incontinence, perifollicular lymphohistocytic infiltrate, fibrosis, and mild perivascular lymphohistocytic infiltrate (Figures 4-5]. 

Colloidal iron stain showed mucin limited to the papillary dermis, however, no basement membrane thickening. Unfortunately, perilesional Direct Immunofluorescence (DIF) was not performed. Based on clinicopathological correlation the diagnosis of old lichen planus pigmentosus following BL was made. Treatment with tacrolimus 0.1% and mometasone resulted in minimal improvement of pigmentation.

## Discussion

Lichen planus pigmentosus commonly affects Indians, Asians, and Middle Eastern populations [1]. It presents as slate grey to dark brown pigmented macules involving mainly the head and neck or the trunk, flexures, and extremities [7]. Lichen planus pigmentosus can follow linear, zosteriform, segmental, and rarely blaschkoid patterns and had been reported as early as 10 years of age [8-11].

Here we present a case of lichen planus pigmentosus following BL with positive ANA as a solitary immunological finding and no clinical evidence of connective tissue disease or any other autoimmune non-rheumatological diseases. Lichen planus and lupus erythematosus (LE) have been infrequently reported in the same patient and have been called the lichen planus-lupus erythematosus overlap [7]. This presentation is unique in several aspects including the distribution of lesions over BL that is rarely encountered, and the immunological association with positive ANA, and lack of any evidence of lupus in the current time of presentation. Having the lesions following abruptio placenta may have triggered an immunological reaction and resulted in this peculiar presentation. Of interest, lichen planus has been reported with pregnancy [8,12].

Differential diagnosis of this presentation includes post-inflammatory hyperpigmentation (PIH) and ashy dermatosis [13]. Usually, the absence of any history of preceding erythema rules out PIH. Commonly ashy dermatosis presents with former lacking erythematous border. Lichen planus pigmentosus and ashy dermatosis are considered two different diseases based on the clinical presentation [13,14]. The findings of hyperkeratosis, hyper granulosis, lichenoid infiltrate, perifollicular infiltrate and fibrosis, and superficial dermal pigment incontinence favor the diagnosis of lichen planus pigmentosus. Vacuolization of basal cells was more common in ashy dermatosis as reported by Rutnin et al. [13].

Based on history and histopathological findings in our patient, the diagnosis of lichen planus pigmentosus was confirmed and the diagnosis of ashy dermatosis and lupus was ruled out.

Usually, lichen planus pigmentosus runs a longer course than the other variants of lichen planus. Successful treatment has been achieved using topical steroids and tacrolimus ointment but with poor response to chloroquine [14]. Small dose isotretinoin and sunscreens showed promising results in controlling the disease progression and extent of pigmentation [15]. Other treatment modalities such as colchicine, dapsone, and mycophenolate mofetil were unsuccessful [15]. Tranexamic acid is one of the modalities that could be used with variable outcomes [16]. Low-fluence Q-switched Nd-YAG (1064 nm) combined with 0.1% tacrolimus, was reported to clear facial pigmentation [17].

## Conclusions

In conclusion, lichen planus pigmentosus in blaschkoid pattern had been rarely reported. According to the timely review of the literature, few cases had been reported so far, and hence the significance of such a case to be added to the current literature. On the other hand, the appearance of the lesions after abruptio placentae stands as a peculiar concurrent event with such a presentation. This event may have triggered the immune response against mosaic keratinocytes and the finding of positive ANA. Follow-up of such cases is required to rule out the progression to systemic lupus or any other connective tissue disease.
